# Mannan-Binding Lectin Inhibits *Candida albicans-*Induced Cellular Responses in PMA-Activated THP-1 Cells through Toll-Like Receptor 2 and Toll-Like Receptor 4

**DOI:** 10.1371/journal.pone.0083517

**Published:** 2013-12-31

**Authors:** Mingyong Wang, Fanping Wang, Jianbin Yang, Dongfang Zhao, Hongpo Wang, Feng Shao, Wenjun Wang, Ruili Sun, Mingzhi Ling, Jingjing Zhai, Shijun Song

**Affiliations:** 1 Department of Laboratory Medicine, Xinxiang Medical University, Xinxiang, China; 2 Department of Clinical Laboratory, The First Affiliated Hospital of Xinxiang Medical University, Xinxiang, China; 3 Department of Clinical Laboratory, The Third Affiliated Hospital of Xinxiang Medical University, Xinxiang, China; 4 Cancer Research Institute, Southern Medical University, Guangzhou, China; Louisiana State University, United States of America

## Abstract

**Background:**

*Candida albicans* (*C. albicans*), the most common human fungal pathogen, can cause fatal systemic infections under certain circumstances. Mannan-binding lectin (MBL),a member of the collectin family in the C-type lectin superfamily, is an important serum component associated with innate immunity. Toll-like receptors (TLRs) are expressed extensively, and have been shown to be involved in *C. albicans*-induced cellular responses. We first examined whether MBL modulated heat-killed (HK) *C. albicans*-induced cellular responses in phorbol 12-myristate 13-acetate (PMA)-activated human THP-1 macrophages. We then investigated the possible mechanisms of its inhibitory effect.

**Methodology/Principal Finding:**

Enzyme-linked immunosorbent assay (ELISA) and reverse transcriptasepolymerase chain reaction (RT-PCR) analysis showed that MBL at higher concentrations (10–20 µg/ml) significantly attenuated *C. albicans*-induced chemokine (e.g., IL-8) and proinflammatory cytokine (e.g., TNF-α) production from PMA-activated THP-1 cells at both protein and mRNA levels. Electrophoretic mobility shift assay (EMSA) and Western blot (WB) analysis showed that MBL could inhibit *C. albicans*-induced nuclear factor-κB (NF-κB) DNA binding and its translocation in PMA-activated THP-1 cells. MBL could directly bind to PMA-activated THP-1 cells in the presence of Ca^2+^, and this binding decreased TLR2 and TLR4 expressions in *C. albicans*-induced THP-1 macrophages. Furthermore, the binding could be partially inhibited by both anti-TLR2 monoclonal antibody (clone TL2.1) and anti-TLR4 monoclonal antibody (clone HTA125). In addition, co-immunoprecipitation experiments and microtiter wells assay showed that MBL could directly bind to the recombinant soluble form of extracellular TLR2 domain (sTLR2) and sTLR4.

**Conclusions/Significance:**

Our study demonstrates that MBL can affect proinflammatory cytokine and chemokine expressions by modifying *C. albicans-*/TLR-signaling pathways. This study supports an important role for MBL on the regulation of *C. albicans*-induced cellular responses.

## Introduction

*Candida albicans* (*C. albicans*) is, a dimorphic fungus, the most common pathogen of humans among fungi and a component of the normal microflora of skin, mucosa and alimentary tract of the healthy host [1]. However, when immune defenses are compromised or the normal microflora balance is disrupted, *Candida* transforms itself into an opportunistic pathogenic killer. Indeed, dissemination of *Candida* is the leading cause of invasive fungal disease in diabetics, premature infants, and surgical patients and of oropharyngeal disease in AIDS patients [2], [3], [4]. *C. albicans* exhibits the ability to grow in a variety of reversible morphological forms [yeast forms (Y), pseudohyphal forms, and hyphal forms (H)] in response to various environmental signals [5]. The ability of *C. albicans* to switch its mode of growth has been shown to be required for the pathogenicity of this fungus [6]. Hyphae formation from yeast cells is a virulence trait enabling this fungus to invade host tissues [7].

The innate immune system, including the complement system, acts as a first line of defense against pathogens. Mannan-binding lectin (MBL), a member of the collectin family in the C-type lectin superfamily, is an important serum component in innate immunity [8], [9]. MBL recognizes a wide range of infectious agents, such as yeasts, bacteria, viruses, parasites, etc. via its C-terminal carbohydrate-recognition domains (CRDs). On binding to pathogens, MBL activates the complement system in an antibody- and C1-independent process involving MBL associated serine proteases, thereby facilitating pathogen removal [10]. MBL also facilitates phagocytosis of cellular debris and may therefore prevent autoimmunity [11], [12].

It is well known that pattern-recognition receptors, such as toll-like receptors (TLRs), play a key role as they can recognize pathogens and activate the acquired immune response [13], [14]. Activating the most TLRs leads to recruitment of MyD88, which can interact with IL-1 receptor-associated kinase, leading to initiation of a signal transduction cascade culminating in nuclear translocation of nuclear factor-κB (NF-κB) family members and then altered the gene expressions, such as IL-8, TNF-α and other cytokines that play important role in immune response and inflammation [15], [16]. Studies have demonstrated the crucial involvement of TLRs in the recognition of fungal pathogens such as *C. albicans*, although less is known regarding the function of these receptors following *Candida* infection [17], [18]. Among the TLR family, mainly TLR2 and TLR4, are involved in the host interaction with *C. albicans* and play a significant role in the development of host immune responses during candidiasis [19]. The host's response to infection is most likely due to the different recognition/activation patterns of these receptors and the release of several proinflammatory cytokines and chemokines [15], [20].

The role of MBL as a modulator of infection appears to be complex and, accordingly, its mechanism of action remains incompletely characterized. Recently, we have proved that MBL can regulate dendritic cell (DC) maturation and cytokine production induced by lipopolysaccharide (LPS) [21], and, MBL can bind to TLR4 directly, and suppress LPS-induced inflammatory cytokine secretion from THP-1 cells [22]. It has also been reported that MBL can facilitate opsonophagocytosis of yeasts [23], and play a crucial role in innate immunity against yeast by enhanced complement activation [24]. Furthermore, MBL deficiency influences innate and antigen-presenting functions of blood myeloid DCs [12]. In addition, recombinant human MBL has utility in the treatment of candidal vaginitis [25]. To date, however, scarce knowledge has been obtained about its role in the regulation of host immune response and the potential mechanism induced by *C. albicans*. As TLRs, mainly TLR2 and TLR4, play critical roles in recognition and signaling of *C. albicans*, it is very informative to investigate how the member of collectin family regulates *C. albicans*/TLR-mediated biological functions.

## Results

### Purification of human MBL

**Figure 1:**
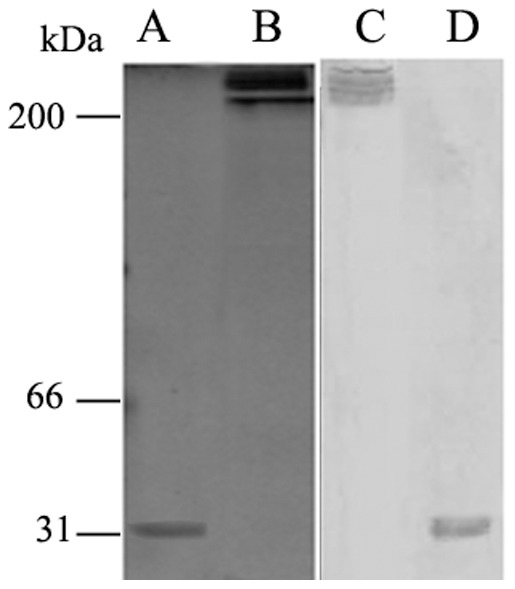
SDS-PAGE and WB analysis of the purified MBL protein. The purified proteins were separated on a 12.5% gel under reducing or non-reducing conditions: (A) SDS-PAGE analysis of the purified MBL under reducing conditions; (B) SDS-PAGE analysis of the purified MBL under non-reducing conditions; (C) WB analysis of the purified MBL under non-reducing conditions; (D) WB analysis of the purified MBL under reducing conditions.

Purified MBL was analyzed by SDS-PAGE and WB (Figure 1). Under reducing conditions, a major band of monomeric MBL with a molecular mass of 31 kDa was detected (Figure 1, A, and D). Under non-reducing conditions, there were several bands with a molecular mass of more than 200 kDa (Figure 1, B, and C). Purified MBL was highly bioactive, as demonstrated by a ligand-binding assay and yeast coagulation assay (data not shown).

### MBL Suppresses *C. albicans* -induced IL-8 and TNF-α production in PMA-activated THP-1 cells

**Figure 2:**
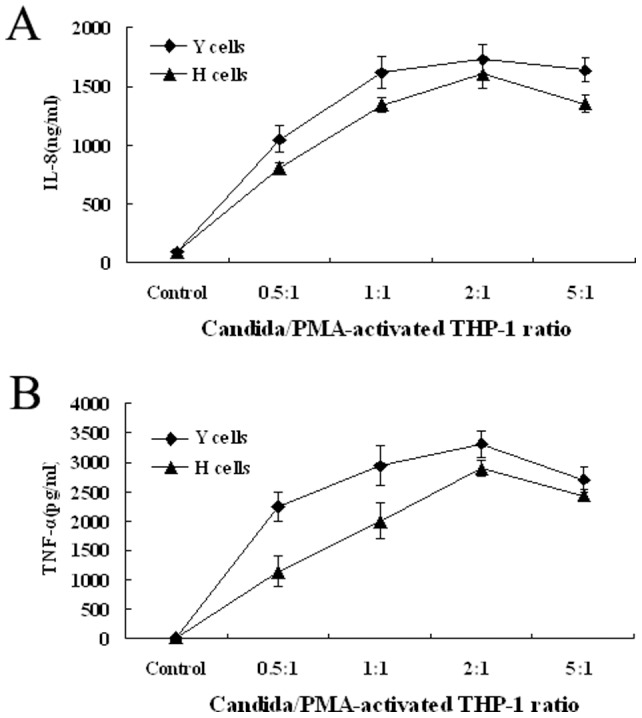
Dose-response IL-8 and TNF-α production by PMA-activated THP-1 cultures stimulated with Y or H cells of *C. albicans*. PMA-activated THP-1 cells (10^6^/ml) were co-cultured with HK Y or H cells of *C. albicans* at the indicated ratios. Cytokines released in culture supernatants after an 18 h incubation were measured by ELISA. Negative controls, PMA-activated THP-1 cells only.

**Figure 3:**
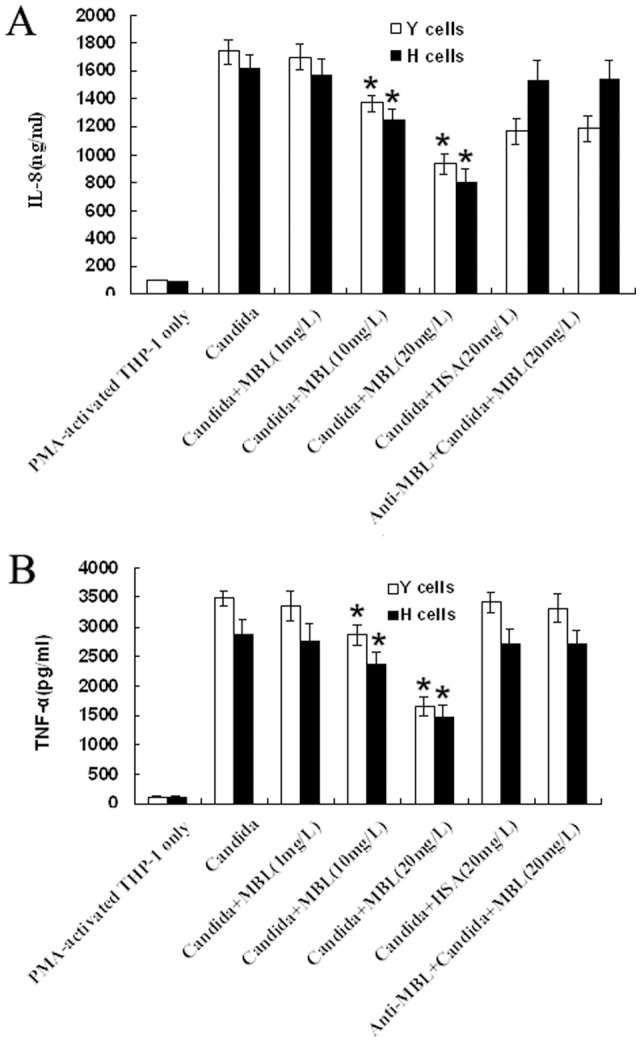
MBL inhibits *C. albicans*-induced IL-8 and TNF-α productions from PMA-activated THP-1 cells. PMA-activated THP-1 cells were stimulated with HK Y or H cells of *C. albicans* at a Candida/THP-1 ratio of 2:1 in the presence of the indicated concentrations of MBL, anti-MBL pAb and MBL, or HSA for 24 h. Supernatants were harvested and subjected to ELISA for IL-8 (A) and TNF-α (B). The data are expressed as mean ± SD of four independent experiments. *P<0.05 as compared to *C. albicans*-stimulated group. Similar results were observed in four independent experiments.

We preliminarily examined whether HK Y or H cells of *C. albicans* could induce production of IL-8 and TNF-α by human PMA-activated THP-1 macrophages. We noticed that PMA-activated THP-1 cells stimulated with HK Y or H cells of *C. albicans* at the indicated ratios exhibited maximal response to secrete IL-8 and TNF-α (Figure 2). We then investigated whether MBL could affect cytokines and chemokines production by THP-1 macrophages stimulated with HK *C. albicans* cells. PMA-activated THP-1 cells were stimulated with HK Y or H cells of *C. albicans* at a Candida/THP-1 ratio of 2:1 in the presence of different concentrations (0, 1, 10, 20 µg/ml) of MBL, 20 µg/ml of human serum albumin (HSA) as control for 24 h, and the supernatants were harvested. The concentration of IL-8 and TNF-α was measured by ELISA. The inductions of IL-8 and TNF-α in PMA-activated THP-1 cells by HK *C. albicans* cells were strongly inhibited by MBL at higher concentrations a dose-dependent manner (10–20 µg/ml), compared with the corresponding PMA-activated THP-1 cells without MBL treatment (*P*<0.05), but low-concentration MBL treatment (1 µg/ml) had no such effect. Inclusion of anti-MBL pAb during the preincubation of the cells with MBL restored the secretion of IL-8 and TNF-α, indicating that the inhibitory effect of MBL is specific (Figure 3).

### MBL attenuates IL-8 and TNF-α mRNA levels

**Figure 4:**
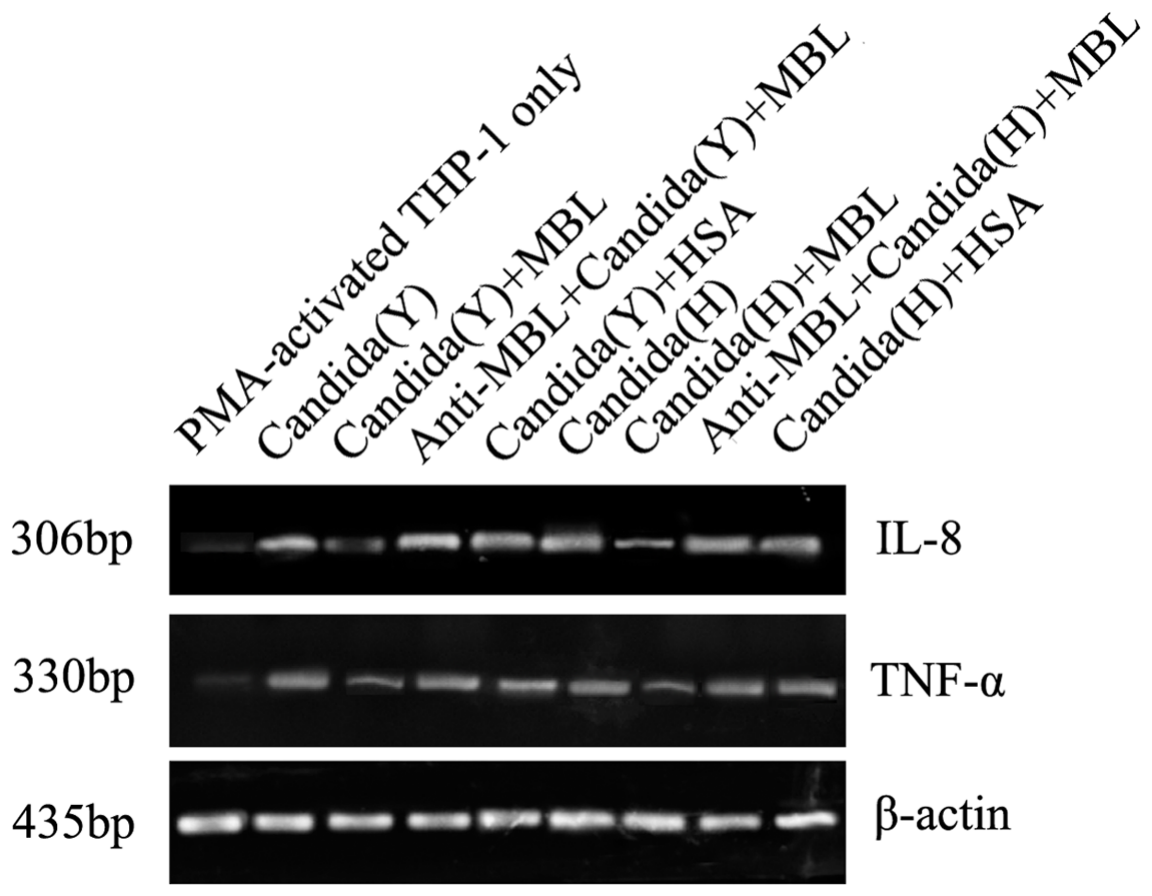
MBL inhibits *C. albicans*-induced IL-8 and TNF-α mRNA expressions in PMA-activated THP-1 cells. PMA-activated THP-1 cells were stimulated with HK Y or H cells of *C. albicans* at a Candida/THP-1 ratio of 2:1 in the presence of the indicated concentrations of MBL, anti-MBL pAb and MBL, or HSA for 24 h. Samples were taken for RNA extraction from various groups, and the amplification and electrophoresis for IL-8, TNF-α genes were carried out at the same time. As an internal control, β-actin was used. These data are representative of four independent experiments.

RT-PCR analysis indicated that IL-8 and TNF-α gene expressions were strongly induced by HK Y or H cells stimulation, but theirs levels were down-regulated significantly in the presence of MBL at higher concentration (20 µg/ml), compared with the corresponding groups that were not treated with MBL(*P*<0.05). Additionally, HSA (20 µg/ml) treatment had no such effect. Inclusion of anti-MBL pAb, while these cells were pre-incubated with MBL, restored the gene levels of IL-8 and TNF-α, suggesting a specific inhibitory effect caused by MBL (Figure 4). These findings suggest that gene expression of IL-8 and TNF-α in HK *C. albicans*-induced THP-1 macrophages could be inhibited by MBL at higher concentrations.

### MBL inhibits NF-κB DNA binding and its translocation in PMA-activated THP-1 cells

**Figure 5:**
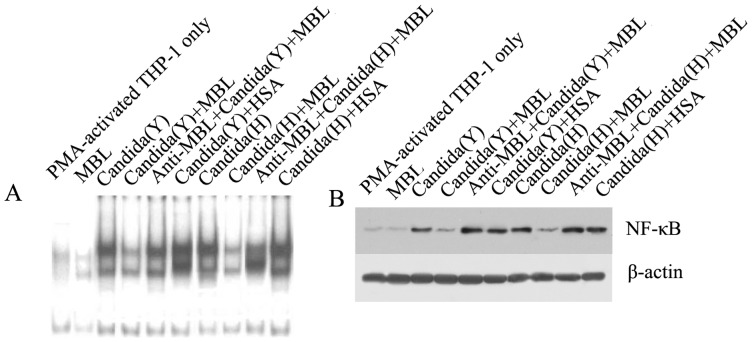
MBL decreases *C. albicans*-stimulated NF-κB binding activity and translocation in PMA-activated THP-1 cells. (A) MBL inhibits *C. albicans*-stimulated NF-κB DNA-binding activity. PMA-activated THP-1 cells (5×10^5^ cells/sample) were stimulated with heat-inactivated Y or H cells of *C. albicans* at a Candida/THP-1 ratio of 2:1 in the presence of the indicated concentrations of MBL, anti-MBL pAb and MBL, or HSA for 1 h, then harvested to prepare nuclear extracts. The nuclear extracts were mixed with radiolabeled NF-κB oligonucleotide probe and analyzed with EMSA. (B) NF-κB translocation is inhibited by MBL in PMA-activated THP-1 cell. PMA-activated THP-1 cells (5×10^5^ cells/sample) were stimulated with HK Y or H cells of *C. albicans* at a Candida/THP-1 ratio of 2:1 in the presence of 20 µg/ml MBL, anti-MBL pAb and MBL, or HSA for 1 h, then the cells were harvested to prepare nuclear extracts. The proteins in the nuclei-free supernatants were separated by 10% SDS-PAGE, followed by transfer to a nitrocellulose membrane. After blocking, the membrane was incubated with the NF-κB-specific mouse anti-human mAb p65, followed by HRP-conjugated secondary antibody. ECL was used to visualize the protein bands. β-actin was used as an internal control.

To analyze whether MBL could modulate *C. albicans*-induced signaling, we examined the effects of MBL on HK *C. albicans*-induced NF-κB activation in PMA-activated THP-1 cells, using two methods: electrophoretic mobility shift assay (EMSA) and western blotting. For EMSA, when nuclear extracts from HK Y or H cells -stimulated THP-1 macrophages were incubated with p40 NF-κB probe, NF-κB binding was enhanced within 1 h after HK Y or H cells -stimulation, and MBL (20 µg/ml) reduced the DNA binding activity; DNA binding was restored by including anti-MBL pAb (Figure 5A). This binding was specific because it could be competed away with a 100-fold excess of unlabeled consensus NF-κB oligonucleotide (data not shown). For WB, analysis of corresponding nuclear fractions by using anti-p65 mAb proved that HK Y or H cells of *C. albicans* significantly increased NF-κB translocation from the cytoplasm to the nucleus in PMA-activated THP-1 cells, and treatment with MBL (20 µg/ml) strongly inhibited this effect. Inclusion of anti-MBL pAb restored NF-κB translocation from the cytoplasm to the nucleus (Figure 5B), further indicating the specific inhibitory effect of MBL on the interaction between *C. albicans* and PMA-activated THP-1 cells.

### Blocking TLR2 or TLR4 inhibits *C. albicans* -induced IL-8 and TNF-α secretion in PMA-activated THP-1 cells

**Figure 6:**
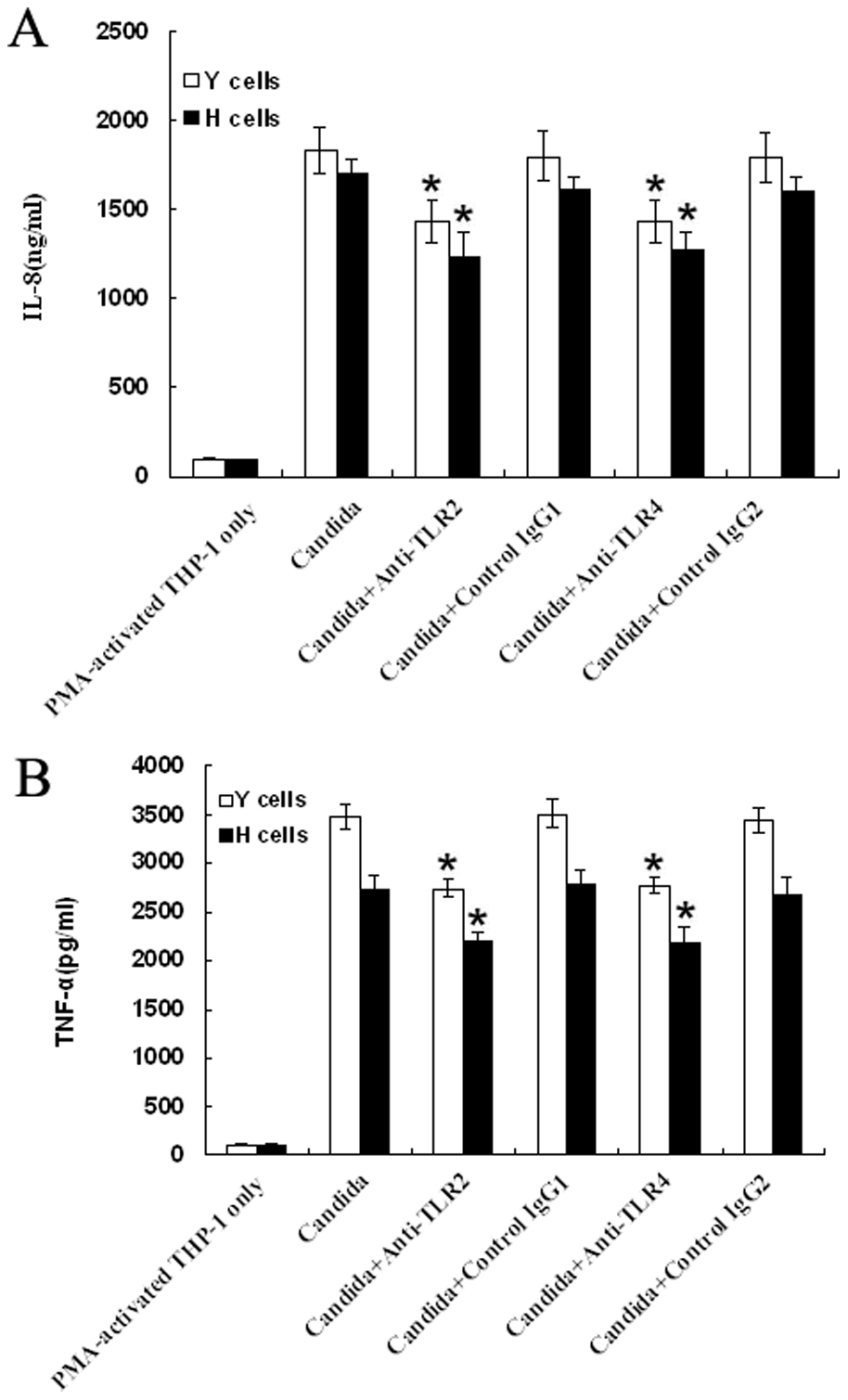
Blocking TLR2 or TLR4 inhibits *C. albicans* -induced IL-8 and TNF-α secretion in PMA-activated THP-1 cells. PMA-activated THP-1 cells were incubated with anti-TLR2 mAb or mouse isotype IgG1 (as a control), anti-TLR4 mAb or mouse isotype IgG2 (as a control), respectively. After 1 h of incubation, cells were stimulated with HK Y or H cells of *C. albicans* at a Candida/THP-1 ratio of 2:1 for 24 h. Supernatants were harvested and subjected to ELISA for IL-8 (A) and TNF-α (B). The data are expressed as mean ± SD of four independent experiments. *P<0.05 as compared to *C. albicans*-stimulated group. Similar results were observed in four independent experiments.

It is known that pattern-recognition receptors, such as TLRs, play a critical role as they recognize pathogens [13], . Among the TLR family, mainly TLR2 and TLR4, are involved in the host interaction with *C. albicans* and play a significant role in the development of host immune responses during candidiasis [19]. Therefore, we assessed the effect of TLR2 and TLR4 in the *C. albicans-* induced model. Incubation of PMA-activated THP-1 cells with anti-TLR2 (clone TL2.1) or anti-TLR4 Ab (clone HTA125) before treated with HK Y or H cells of *C. albicans* at the indicated ratios, the supernatants were harvested. The concentration of IL-8 and TNF-α was measured by ELISA. The experiment results showed that both anti-TLR2 mAb and anti-TLR4 mAb could partially suppress IL-8 (Figure 6A) and TNF-α (Figure 6B) production from *C. albicans* -induced THP-1 macrophages independently, whereas the isotype control had no effect. Together, these data suggest that *C. albicans* induced cellular responses in PMA-activated THP-1 cells is through TLR2 and TLR4.

### MBL attenuates *C. albicans* -induced TLR2 and TLR4 expression in PMA-activated THP-1 cells

**Figure 7:**
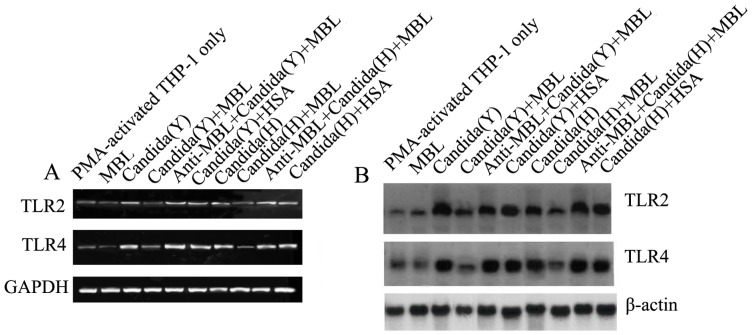
MBL inhibits *C. albicans*-induced TLR2 and TLR4 expressions in PMA-activated THP-1 cells. (A) Expression of TLR2 and TLR4 in PMA-activated THP-1 cells was analyzed by RT-PCR. Total RNA was isolated from PMA-activated THP-1 cells cultured in medium with or without HK Y or H cells of *C. albicans* at a Candida/THP-1 ratio of 2:1 for 2 h by using a Qiagen kit. Glyceraldehyde-3-phosphate dehydrogenase (GAPDH) was used as an internal control. (B) Northern blot analysis. Total RNA was prepared from PMA-activated THP-1 cells cultured in medium (with or without HK Y or H cells of *C. albicans* at a Candida/THP-1 ratio of 2:1) for 2 h, Blots were hybridized with probes specific for TLR2 or TLR4. β-actin was used as an internal control.

We have proved that MBL could bind to TLR4 directly, and suppress LPS-/TLR-mediated biological function [22]. TLR4 is the binding site (ligand) for MBL expressed on THP-1 cells. As it was found that MBL could modulate *C. albicans*-induced cellular responses, and TLR2 and TLR4 are involved in the host interaction with *C. albicans*, it is very informative to investigate whether TLR2 and TLR4 are involved in the regulative effect of MBL modulating *C. albicans*-induced cellular responses. We stimulated PMA-activated THP-1 cells for 24 h with HK Y or H cells of *C. albicans* and then examined their RNA expression using two methods: RT-PCR and Northern blot. As shown in Figure 7(A and B), TLR2 and, TLR4 mRNA gene expressions were strongly induced by HK Y or H cells stimulation, but theirs levels were down-regulated significantly in the presence of MBL at higher concentration (20 µg/ml), compared to the corresponding groups without MBL treatment, but not by HSA (20 µg/ml). Inclusion of anti-MBL pAb during the preincubation of the cells with MBL restored the genes levels of TLR2 and TLR4, indicating the specific inhibitory effect of MBL. All these data suggest that MBL at higher concentrations can inhibit *C. albicans*-induced TLR2 and TLR4 mRNA expressions.

### Inhibition of binding of MBL to PMA-activated THP-1 cells by anti-TLR2 mAb and anti-TLR4 mAb

**Figure 8:**
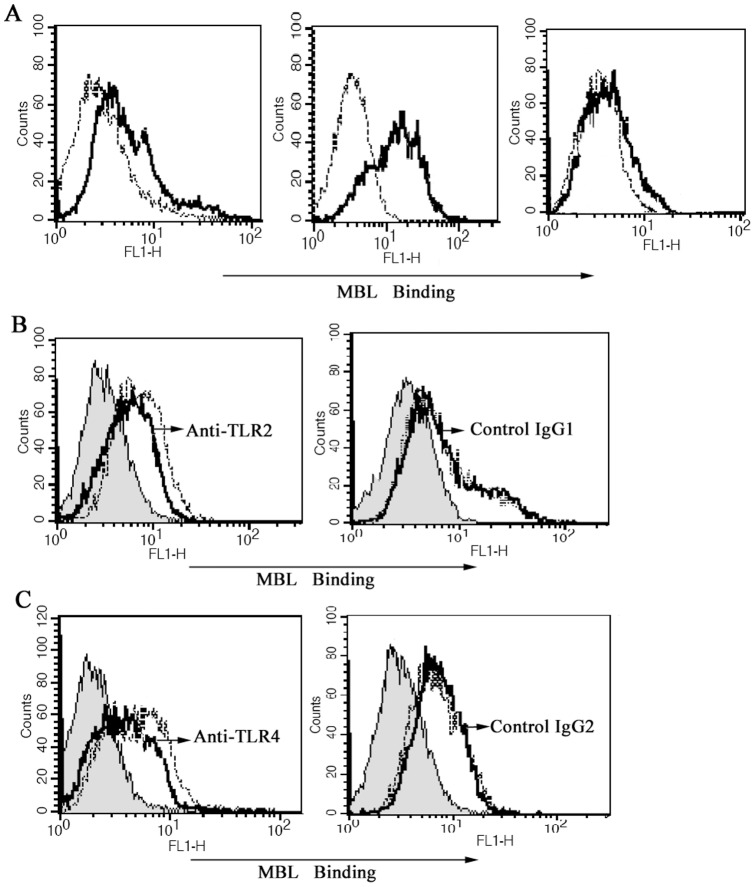
Inhibition of binding of MBL to PMA-activated THP-1 cells by anti-TLR2 mAb and anti-TLR4 mAb. (A) MBL binds to PMA-activated THP-1 cells in a Ca^2+^-dependent manner. PMA-activated THP-1 cells were incubated with biotinylated MBL in Tris-buffered saline solution containing various concentration of calcium ion. (a) Tris-buffered saline with 1.3 mM CaCl_2_; (b) Tris-buffered saline with 5 mM CaCl_2_; (c) Tris-buffered saline with 5 mM EDTA. Each solution of THP-1 cells was incubated for 30 min at 37°C, binding of MBL to the cells was analyzed by FCM, as shown in the representative histograms. Black lines, biotinylated MBL binding. Dotted lines, negative controls (the cells only). (B) Anti-TLR2 mAb attenuates the binding of MBL to PMA-activated THP-1 cells. PMA-activated THP-1 cells were incubated with anti-TLR2 mAb or control mouse isotype IgG1, for 10 min before the addition of biotinylated MBL, and the binding of MBL was revealed by ExtrAvidin-FITC. Shaded curves, cells only, black solid line, with anti-TLR2 mAb, dotted line, without anti-TLR2 mAb. Data shown are representatives of four independent experiments. (C) Anti-TLR4 mAb attenuates the binding of MBL to PMA-activated THP-1 cells. PMA-activated THP-1 cells were incubated with anti-TLR4 mAb or control mouse isotype IgG2, for 10 min before the addition of biotinylated MBL, and the binding of MBL was revealed by ExtrAvidin-FITC. Shaded curves, cells only, black solid line, with anti-TLR4 mAb, dotted line, without anti-TLR4 mAb. Data shown are representative of four independent experiments.

To further study the possible mechanism of MBL's effect on the interaction between HK *C. albicans* cells and PMA-activated THP-1 cells, we sought to investigate whether TLR2 and TLR4 are the binding site (ligand) for MBL expressed on PMA-activated THP-1 cells. The experiment results showed that MBL could directly bind to PMA-activated THP-1 cells in the presence of Ca^2+^ (Figure 8A). Furthermore, not only anti-TLR4 mAb (Figure 8B) but also anti-TLR4 mAb (Figure 8C) could partially inhibit the binding of MBL to the cells independently, indicating that both TLR2 and TLR4 are the binding site (ligand) for MBL expressed on PMA-activated THP-1 cells.

### Binding of MBL to TLR2 and TLR4

**Figure 9:**
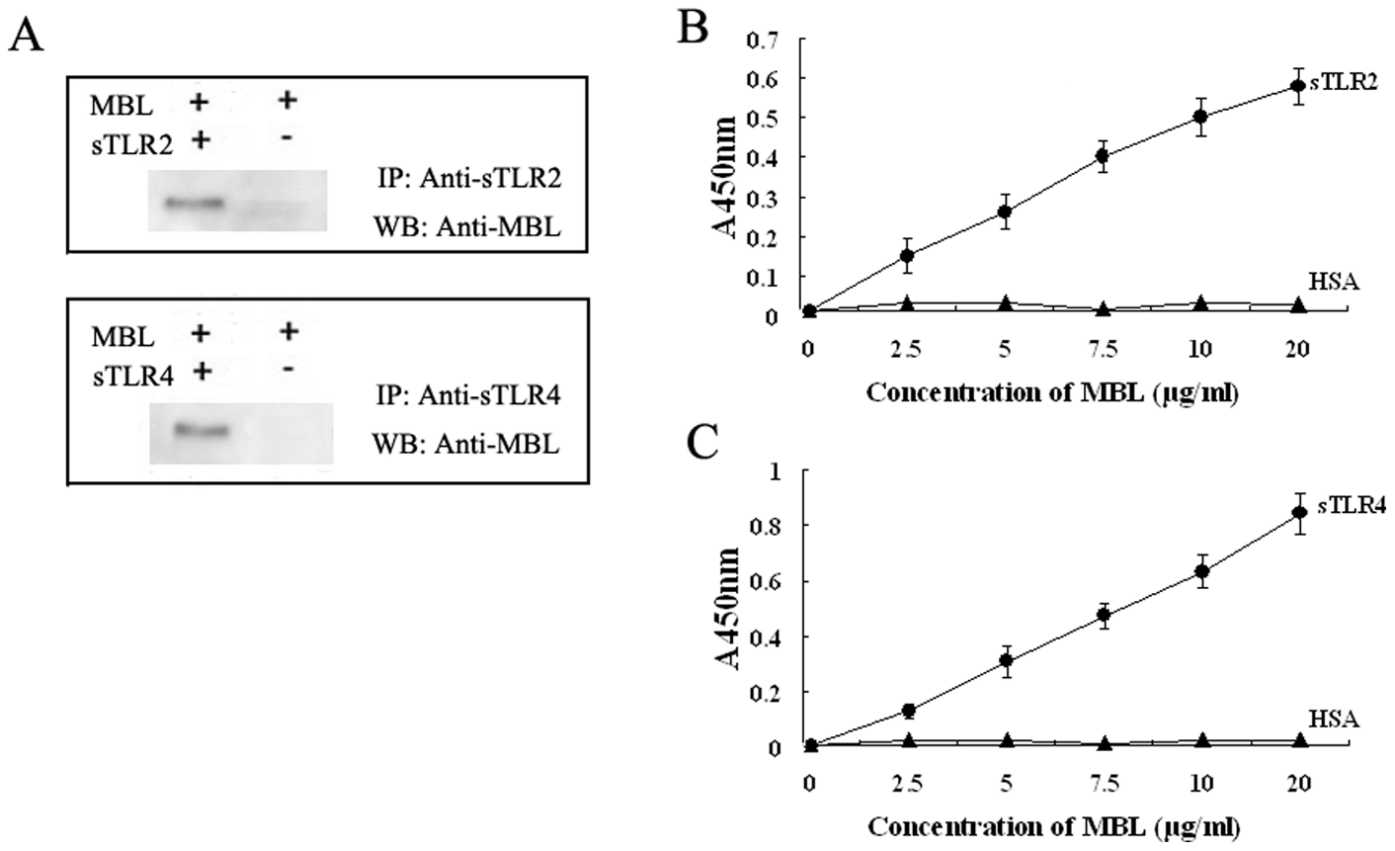
MBL binds to sTLR2 and sTLR4. (A) 2 µg of MBL were incubated with sTLR2 or sTLR4 followed by incubation with anti-sTLR2 pAb or anti-sTLR4 pAb, respectively. The immune complexes were precipitated with protein G-Sepharose and subjected to SDS-PAGE under reducing conditions. The proteins on the gel were transferred to polyvinylidene difluoride membrane and immunoprobed with anti-human MBL monoclonal antibody as described under “Materials and Methods” IP, immunoprecipitate; WB, Western blot. sTLR2 (B) or sTLR4 (C) was coated onto microtiter wells and incubated with the indicated concentrations of MBL, respectively. The binding of MBL to sTLR2 and sTLR4 was detected by anti-MBL mAb, as described under “Materials and Methods”. The data shown are mean ± S.E. of three experiments.

To further actually demonstrate an interaction between MBL and TLRs, it should be interesting to examine this interaction at the molecular level using co-immunoprecipitation experiments with anti-MBL and anti-TLRs antibodies. When MBL and deglycosylated sTLR2 were coincubated, MBL was associated with sTLR2 that was immunoprecipitated with anti-sTLR2 antibody (Figure 9A, *upper panel*). Similarly MBL was clearly coprecipitated with deglycosylated sTLR4 (Figure 9A, *lower panel*). The results demonstrate that MBL could bind to sTLR2 and sTLR4 directly in the solution phase. We further examined the binding of MBL to sTLR2 and sTLR4 coated onto microtiter wells. As shown in Figure 9(B and C), MBL bound to the solid phase sTLR2 and sTLR4 in a concentration-dependent manner. From these results, we conclude that MBL binds to the extracellular domain of TLR2 and TLR4.

## Discussion

MBL, as well as surfactant protein A (SPA), surfactant protein D (SPD), and ficolin, are members of a unique family of proteins, known as the defense collagens. It is a major soluble pattern-recognition receptor in the innate immune system. Innate immune responses play a crucial role in host defence against *C. albicans*. Monocytes and monocyte-derived macrophages activate and regulate innate immune responses through the interaction between PRPs expressed on their cellular membranes and the polysaccharide components from cell wall of *C. albicans* [26], [27].

In this study, we investigated how the soluble pattern recognition molecule regulated *C. albicans-*induced immune responses of human PMA-activated THP-1 macrophages. Unlike other leukemic cell lines, THP-1 as a leukemic cell line derived from a patient with acute monocytic leukemia has no chromosomal abnormalities [28]. It has lost some characteristics of normal monocytes/macrophage, so the experimental data obtained directly from THP-1 cell line could not reflect the actions between *C. albicans* and phagocytes. Therefore, it is necessary to induce the differentiation of naïve THP-1 cell line into the macrophage using PMA before the stimulation with *C. albicans.* Our experimental data demonstrated that MBL at higher concentrations (10–20 µg/ml), but not at lower concentration (1 µg/ml), significantly inhibited *C. albicans*-induced IL-8 and TNF-α production at both protein and gene levels in PMA-activated THP-1 macrophages, suggesting MBL could modify *C. albicans*-induced cytokines and chemokines secretion.

TLRs constitute a family of pattern-recognition receptors (PRRs) that recognize molecular signatures of microbial pathogens and function as sensors for infection that induce the activation of the innate immune responses as well as the subsequent development of adaptive immune responses. Among the TLR family, TLR2 and TLR4 are expressed very extensively (e.g., monocytes, macrophages, immature DCs, T cells and B cells), and play critical roles in recognition and signaling of *C. albicans*. Recognition of *C. albicans* by TLR2 and TLR4 on the monocytes activates intracellular signaling pathways that trigger production of proinflammatory cytokines and chemokines that are critical for innate host defence and orchestrate the adaptive response [29]. In this study, we also assessed the effect of TLR2 and TLR4 in the *C. albicans-* induced model by using anti-TLR2 and anti-TLR4 Ab. The experiment results showed that both anti-TLR2 mAb and anti-TLR4 mAb could partially suppress IL-8 and TNF-α production from *C. albicans* -induced THP-1 macrophages independently. All researching results further proved that *C. albicans* induced cellular responses of PMA-activated THP-1 cells were through TLR2 and TLR4.

MBL has long been known to recognize pathogens or autologous apoptotic cells via its CRD and to interact with autologous cells via its collagen-like region (CLR). It was believed that the CRD does not interact with normal autologous cells, but recently Downing *et al*. [30] reported calcium-dependent MBL binding to autogeneic B lymphocytes, monocytes and imMDCs via its C-type lectin-binding site. More recently, we also proved that MBL bound to human THP-1 monocytes in calcium-dependent manner [22]. To study the possible mechanism of MBL's effect on the interaction between HK *Candida* cells and PMA-activated THP-1 cells, the experiments of MBL binding to PMA-activated THP-1 cells were performed. Our data significantly demonstrated that MBL could directly bind to PMA-activated THP-1 cells in a calcium-dependent manner and lead to less NF-κB activation by *C. albicans*, what leads to less mRNA synthesis and less protein production. In addition, the binding can be partially inhibited by both anti-TLR2 monoclonal antibody (clone TL2.1) and anti-TLR4 monoclonal antibody (clone HTA125), indicating that not only TLR2 but also TLR4 are the binding site (ligand) for MBL expressed on PMA-activated THP-1 cells. To actually demonstrate an interaction between MBL and TLRs, we prepared a soluble form of recombinant extracellular TLR2 domain (sTLR2) and sTLR4, and co-immunoprecipitation experiments and microtiter wells assay were used to evaluate the binding of MBL to sTLR2 or sTLR4. The experiment results showed that MBL could directly interact with sTLR2 and sTLR4. All these suggest that the ability of MBL is versatile in immune regulation.

To further investigate the possible mechanism, we examined the transcript levels of TLR2 and TLR4. Our data clearly demonstrated that expressions of TLR2 and TLR4 in PMA-activated THP-1 cells could be induced by both Y cells and H cells. These expressions induced with HK *C. albicans* were profoundly inhibited by MBL at higher concentrations (10∼20 mg/L) but not MBL at lower concentrations (1 mg/L). All these data further prove that MBL regulates *C. albicans*-induced cellular responses of human PMA-activated THP-1 macrophages through TLR2 and TLR4.

It would seem counterintuitive that a protein that inhibits human macrophages function would arise acutely just at the time when vigorous immune stimulation is most needed (one to several days post infection). Normally, this is a desirable homeostatic mechanism that would come into play after sterilizing immunity had been achieved. In addition, it is interesting that Ip *et al*. [31], [32] have reported that MBL could increase signaling from TLR2 and TLR6, and Shimizu *et al*. [33] recently showed that recombinant MBL could interact with TLR4 and MD-2. More recently, we proved that MBL could bind to TLR4 directly, and suppress LPS-/TLR-mediated biological function [22]. The finding of an inhibitory effect of MBL on *C. albicans*-induced immune responses of human macrophages through TLR2 and TLR4 in this study further demonstrated the versatile ability in immune regulation of this member of the collectin family.

Chemokines are chemoattractant cytokines that play crucial roles in host defense against infectious agents by their ability to induce and regulate magnitude and composition of leukocyte influx at the infected site. Furthermore, they may act as direct activators of the antimicrobial activity of leukocytes and also modulate lymphocyte differentiation into predominantly T-helper type 1 (Th1) or Th2 patterns [7], [34]. As one of the most important inflammatory factor, IL-8 has an obvious function of chemotaxis and functional activation on neutrophils, and it can induce chemotaxis of CD4^+^ and CD8^+^ T cells. So far, we have known that IL-8 participates in processes of many diseases and tissue damages, such as polyarthritis destruens, psoriasis vulgaris, glomerulonephritis, and so on. In addition, IL-8 and TNF-α play an important role in the development of cellular- mediated immune response and inflammatory reaction, and they also contribute to the development of autoimmune disease.

Inflammatory reaction is an essential composition of defense to pathogen, however, when it overacts or runs inadequately, harmful or even fatal results may come along. Numerous studies have confirmed that the imbalanced cytokines mediating the interaction of natural and acquired immunity contribute to human sepsis and immune dysfunctions. Overproduction of proinflammatory cytokines and chemokines (e.g. TNF-α, IL-12, IFN-γ and IL-8) results in sustained sepsis, shock, and even death [35], [36]. In this study, higher concentrations of MBL inhibited *C. albicans*-induced secretion of such proinflammatory cytokines and chemokines as TNF-α and IL-8, indicating that MBL might be implicated in the anti-inflammatory effect and immunoregulation, reducing the incidence of shock and prevent endotoxemia-induced death. Therefore, in the acute phase of *C. albicans* infection, MBL may play an important role in balancing the homeostasis and protecting the host from the excessive inflammation and damage. This study paves the way for a novel treatment of inflammatory shock by modulating excessive activated human macrophages with MBL.

### Conclusions

Together, the results presented in this study demonstrated that MBL could attenuate PMA-activated THP-1 macrophages cytokines and chemokines production induced by HK *C. albicans*. MBL could bind directly to PMA-activated THP-1 cells a Ca^2+^-dependent manner and led to less TLR2 and TLR4 expression and less NF-κB activation in PMA-activated THP-1 cells by *C. albicans*. We propose that MBL could affect inflammatory cytokine expression by modulating *C. albicans-*/TLR-signaling pathways. This study supports an important role for MBL in balancing the homeostasis and protecting the host from the excessive inflammation and damage.

## Materials and Methods

### Purification of human MBL

All steps were carried out at 4°C unless stated otherwise. MBL was isolated from human plasma according to Tan et al [37], modified as described [21], [38]. Briefly, Pooled human plasma of 2000 ml from healthy donors (provided by The Third Affiliated Hospital of Xinxiang Medical University, China. This study was conducted in accordance with the declaration of Helsinki. This study was conducted with approval from the Ethics Committee of Xinxiang Medical University. Written informed consent was obtained from all participants) was adjusted to 20 mM CaCl_2_ and allowed to clot upon incubation for 2 h at 37°C and then overnight at 4°C. After extraction and elimination of most of the unrelated proteins, the residual fraction was solubilized. MBL was purified from the extract by a process involving three chromatographic steps. The first step, was affinity chromatography on a mannan-agarose (Sigma, Poole, UK) column, which was the key step of the process. The subsequent steps were anion-exchange chromatography and gelfiltration on a Mono-Q HR 5/5 column (Pharmacia Biotech Europe, Orsay, France) and a Superose 6 HR 10/30 column (Pharmacia Biotech, Orsay, France), respectively. The MBL preparations were determined without endotoxin contamination by Limulus amebocyte lysate assay. The purified MBL was also evaluated by SDS-PAGE and Western blot (WB).

### Purification of fungal cells

*C. albicans* strain ATCC 90028 was maintained on Sabouraud dextrose agar (Difco Laboratories) at 4°C. Yeast cells was grown at 30°C in YPD (2% yeast extract, 1% bactopeptone and 2% glucose) or in GMM (2% glucose and 1× yeast nitrogen base) or GMM supplemented with nutrients. For hyphal growth, the yeast cells were inoculated into YPD +10% serum, Lee's (pH 7.0) medium [39], RPMI-1640 or Spider's medium and grown at 37°C. Solid media contain 1.5% agar. G1 yeast cells were prepared by centrifugal elutriation as described [40]. Y cells or H cells were harvested by centrifugation, washed three times in 0.01 M phosphate buffer (pH 7.2) containing 0.15 M NaCl (PBS) and then heat-killed (HK) at 65°C for 1 h.

### Preparation of a soluble form of recombinant extracellular TLR2 domain (sTLR2) and sTLR4

The recombinant soluble form of TLR2 consisting of the putative extracellular domain (Met1-Arg587) and a His6 tag at the C-terminal end was constructed, and the sTLR2 protein was expressed in the baculovirus-insect cell expression system using the methods described previously [41]. The protein was purified from the medium using a column of nickel-nitrilotriacetic acid beads (Qiagen, Valencia, CA) by a method described previously [42]. Polyclonal Ab against sTLR2 was prepared as described previously [41]. A soluble form of recombinant TLR4 extracellular domain (sTLR4) protein, consisting of the putative extracellular domain (Met1-Lys631) and a 6 His tag at the C-terminal end, was also prepared in our lab as described [43].

For deglycosylation, sTLR2 (4 µg) or sTLR4 was incubated with 2 unit of N-glycosidase F (Roche Diagnostics, IN) at 37°C for 2 h in 10 mM Tris buffer (pH 7.4) containing 10 mM EDTA, 2% (v/v) β-mercaptoethanol, 0.1% (w/v) SDS, and 1% (v/v) Nonidet P-40. The removal of oligosaccharides was confirmed by a carbohydrate detection kit (G. P. Sensor, J-Oil Mills, Yokohama, Japan) according to the manufacturer's instructions.

### PMA-activated THP-1 cultures and cytokine assays

The monocytic human cell line THP-1 (a gift from Dr. J. H. Han, Scripps, La Jolla, CA) was cultured in IMDM growth medium (Gibco BRL Products, Rockville, MD) supplemented with 10% fetal calf serum, 1% penicillin-streptomycin. Cells were maintained in a 5% CO_2_ humidified atmosphere at 37°C. Cell viability was assessed by trypan blue exclusion.

In order to induce the differentiation of naïve THP-1 cell line into the macrophage, THP-1 monocytes were activated by treatment with phorbol 12-myristate 13-acetate (PMA) (1 ng/ml, Sigma) before the stimulation with *C. albicans.* PMA-activated THP-1 cells (1×10^6^/ml) were seeded in 24-well tissue culture plates (Corning-Costar, MA, USA) in IMDM complete medium, and maintained at 37°C in a 5% (v/v) CO_2_ environment for 2 h after MBL was added to a range of final concentrations (0–20 µg/ml). PMA-activated THP-1 cultures, either unstimulated or stimulated with HK Y or H cells of *C. albicans* at the indicated ratios were prepared and incubated overnight at 37°C in a 5% (v/v) CO_2_ environment. The cell free culture supernatants were harvested and the amount of IL-8 and TNF-α was measured by enzyme-linked immunosorbent assay (ELISA) (R&D Systems, Minneapolis, MN, USA) according to the manufacturer's instructions. In control groups, HAS (20 µg/ml) was used. To demonstrate the specificity of the responses to MBL, anti-MBL pAb (R&D systems, MN, USA) was used.

To investigate whether priming could be blocked, we preincubated PMA-activated THP-1 cells for 1 h with 10 µg/ml anti-TLR2 Ab (clone TL2.1, eBioscience, San Diego, CA) or 10 µg/ml anti-TLR4 Ab (clone HTA125, Santa Cruz Biotechnology, Santa Cruz, CA), or with the isotype control of both antibodies (10 µg/ml) respectively, before treated with HK Y or H cells of *C. albicans* at the indicated ratios and incubated for 24 h. Cytokines were measured by ELISA.

### Reverse transcriptasepolymerase chain reaction (RT-PCR) analysis of cytokine mRNA expression in PMA-activated THP-1 cells

PMA-activated THP-1 cells cultures were prepared as described above. Total RNA was extracted with TRIzol and cDNA was synthesized using 2 μg total RNA with SuperScript III reverse transcriptase according to the manufacturer's instructions (Invitrogen, Carlsbad, CA, USA). Real-time PCR was performed using Syber green and a Cepheid Smart Cycler System (Cepheid, Sunnyvale, CA, USA). Primers and programmes were as described [26], [44]: IL-8, sense primer 5′- CTT TGT CCA TTC CCA CTT CTG A -3′ and anti-sense primer 5′-TCC CTA ACG GTT GCC TTT GTA T-3′, amplified product 306 bp, annealing at 55°C, 35 cycles; TNF-α, 5′-AAG CCT GTA GCC CAT GTT GT-3′ and 5′-CAG ATA GAT GGG CTC ATA CC-3′, 330 bp, annealing at 54°C, 29 cycles, and β-actin, 5′-CCA GAG CAA GAG AGG CAT CC-3′ and 5′-GTG GTG GTG AAG CTG TAG CC-3′, 435 bp, annealing at 56°C, 35 cycles. The PCR products were visualized by electrophoresis and ethidium bromide staining, identified by their predicted molecular sizes, and quantitatively evaluated by densitometric scanning.

### Analysis of NF-kB by electrophoretic mobility shift assay (EMSA) and western blotting(WB)

Nuclear extracts were prepared from 5×10^5^ PMA-activated THP-1 cells which were stimulated with HK Y or H cells of *C. albican* at the indicated ratios in the presence of 20 µg/ml MBL, MBL and anti-MBL pAb or HSA for 1 h, respectively. Conditions for EMSA have been described previously [21], .

To detect translocation of NF-kB by WB, The extracts were centrifuged for 10 min at 1200×g and proteins of nucleifree supernatants were separated by 10% SDS-PAGE and transferred to polyvinylidene difluoride membranes (Millipore, Before, MA, USA). Membranes were blocked with PBS containing 0.05% Tween 20 and 5% non-fat dry milk and probed with the NF-κB-specific mouse anti-human mAb p65 (Santa Cruz Biotechnology, CA, USA), then incubated with appropriate secondary antibodies conjugated to horseradish peroxidase. Protein bands were visualized by using Supersignal reagents (Pierce, Rockford, IL, USA).

### RT-PCR and Northern blot analysis of TLR2 and TLR4 expression

Total RNA was extracted and cDNA was synthesized as described above. PCR amplification was performed with Taq gold polymerase (Perkin Elmer, CA). The primers used for RT-PCR were as follows, TLR2: 5′-GCCAAGCTCTCTAAACATTT-3 and 5′-TTTCACTGCTTTCAACTGGTA-3′; TLR4: 5′-TGG ATA CGT TTC CTT ATA AG-3′ and 5′-GAA ATG GAG GCA CCC CTT C-3′; Glyceraldehyde-3-phosphate dehydrogenase (GAPDH): 5′-GGTATCGTCGAAGGACTCATGAC-3′ and 5′-ATGCCAGTGAGCTTCCCGTTCAGC-3′. GAPDH was used as a reference gene for this study.

For Northern blot, total RNA isolated from treated and untreated PMA-activated THP-1 cells was analyzed by electrophoresis through 1% agarose gel and then transferred onto a Hybond-N1 membrane (Amersham Pharmacia Biotech) by a capillary transfer method. The plasmids containing human TLR2, TLR4, or actin cDNAs were labeled with [α-^32^P]dCTP (3,000 Ci/mmol; Amersham Pharmacia Biotech, UK). After a 30-min prehybridization at 65°C, the membranes were hybridized with 10^6^ cpm of the labeled probe/ml for 3 h at 65°C in Rapid hyb buffer (Amersham Pharmacia Biotech) and washed twice with 2× SSC (1× SSC: 0.15 M NaCl, 0.015 M sodium citrate) and 1% sodium dodecyl sulfate (SDS), and finally washed twice with 0.1× SSC at room temperature for 30 min. Membranes were exposed for 4–48 h at –80°C with intensifying screens.

### Analysis of anti-TLR2 and anti-TLR4 mAb's effect on the binding of MBL to PMA-activated THP-1 cells

Washed THP-1 macrophages (2×10^5^) were resuspended in Tris-buffered saline (pH 7.4) containing 5 mM CaCl_2_, 1% bovine serum albumin (buffer A). Three kinds of Tris-buffered saline containing different calcium concentrations were alternatively used for binding assays. For investigation of TLR2 Ab or TLR4 Ab's effect on the binding of MBL to PMA-activated THP-1 cells, the possible inhibitor, anti-TLR2 Ab (clone TL2.1, 10 µg/ml) or anti-TLR4 Ab (clone HTA125, µg/ml), or with the isotype control (10 µg/ml), was preincubated with the cells for 10 min. Each cell suspension (0.2 ml) was first incubated for 30 min on ice with either biotinylated MBL or unlabelled MBL. Then the cells were incubated for 30 min on ice with ExtrAvidin-FITC (Sigma, Madrid, Spain) at a final dilution of 1 in 100. After washing, the cells were analyzed by FACSCalibur.

### Analyses of binding of MBL to sTLR2 and sTLR4

Two methods were used to evaluate the binding of MBL to sTLR2 or sTLR4. One method was used co-immunoprecipitation experiments. Briefly, MBL (2 µg) was incubated with 2 µg of either sTLR2 or sTLR4 at 37°C for 2 h in 20 mM Tris buffer (pH 7.4) containing 0.15 M NaCl, 5 mM CaCl_2_, and 3 mg/ml BSA. The mixture was then incubated with 2µg of anti-sTLR2 pAb or 2 µg of anti-sTLR4 pAb at 4°C for 2 h, and immune complexes were precipitated with protein G-Sepharose (40 µl) by gentle shaking at 4°C for 2 h. The beads were washed three times with 20 mM Tris buffer (pH 7.4) containing 0.15 M NaCl, 5 mM CaCl_2_, and 0.1% (v/v) Triton X-100 and resuspended in SDS sample buffer. The samples were subjected to SDS-PAGE after boiling for 5 min under reducing conditions and transferred to polyvinylidene difluoride membrane. The membrane was immunoprobed with anti-human MBL mAb HYB 131–11 (1∶2000, Abcam, UK) followed by incubation with HRP-conjugated anti- mouse IgG. The proteins that reacted with the antibodies were finally visualized by using a chemiluminescence reagent (SuperSignal, Pierce).

The binding of MBL to sTLR2 and sTLR4 was also examined using microtiter wells. sTLR2 or sTLR4 (10 µg/ml, 50 µl/well) was coated onto microtiter wells. The wells were incubated with 10 mM Hepes buffer (pH 7.4) containing 0.15 M NaCl, 5 mM CaCl_2_, and 5 mg/ml BSA (buffer B) to block nonspecific binding. The indicated concentrations of MBL in buffer B were added and incubated at 37°C for 5 h. In some experiment, 5 mM EDTA was substituted for CaCl_2_ in buffer B. After washing, the wells were incubated with anti-MBL mAb HYB 131-11 (1∶5000). Binding was detected with HRP-labeled goat anti-mouse IgG (1: 1500), and the absorbance at 450 nm was measured. In control groups, HSA was used.

### Statistical analysis

The mean and SD were calculated by Excel software (Microsoft). Student's t-test was used for statistical analysis, and *P*<0.05 was considered significant.
